# A Role for Adenosine A1 Receptors in GABA and NMDA-Receptor Mediated Modulation of Dopamine Release: Studies Using Fast Cyclic Voltammetry

**DOI:** 10.3390/s8095516

**Published:** 2008-09-05

**Authors:** John J. O′Connor, Carmel O′Neill

**Affiliations:** UCD School of Biomolecular and Biomedical Science, UCD Conway Institute of Biomolecular & Biomedical Research, University College Dublin, Belfield, Dublin 4, Ireland

**Keywords:** Fast cyclic voltammetry, Adenosine, GABA, NMDA, dorsolateral striatum

## Abstract

In the striatum many neurotransmitters including GABA, glutamate, acetylcholine, dopamine, nitric oxide and adenosine interact to regulate synaptic transmission. Dopamine release in the striatum is regulated by a number of pre- and post-synaptic receptors including adenosine. We have recently shown using isolated rat striatal slices, and the technique of fast cyclic voltammetry, that adenosine A_1_ receptor-mediated inhibition of dopamine release is modulated by dopamine D_1_ receptors. In the present study we have investigated the influence of NMDA and GABA receptor activation on the modulation of electrically stimulated dopamine release by adenosine. Application of the adenosine A_1_ receptor agonist, N^6^-cyclopentyladenosine (CPA), concentration-dependently inhibited dopamine release to a maxiumum of 50%. Perfusion of the glutamate receptor agonist, NMDA, in low magnesium, caused a rapid and concentration-dependent inhibition of dopamine release. Prior perfusion with the adenosine A_1_ receptor antagonist, DPCPX, significantly reduced the effect of 5 μM and 10 μM NMDA on dopamine release. The GABA_A_ receptor agonist, isoguvacine, had a significant concentration-dependent inhibitory effect on dopamine release which was reversed by prior application of the GABA_A_ receptor antagonist, picrotoxin, but not DPCPX. Finally inhibition of dopamine release by CPA (1μM) was significantly enhanced by prior perfusion with picrotoxin. These data demonstrate an important role for GABA, NMDA and adenosine in the modulation of dopamine release.

## 1. Introduction

The striatum receives many synaptic inputs from many different sources. Glutamatergic afferents arrive from many areas of the cortex and the thalamus, whereas the nigrostriatal pathway and other intrinsic circuits provide the striatum with acetylcholine, GABA, dopamine, nitric oxide and adenosine [1]. All these neurotransmitter systems interact with each other and with voltage-dependent conductances to regulate the efficacy of synaptic transmission within the striatum [2]. Complex interations have now been clearly shown for adenosine A_1_ & dopamine D_1_ and adenosine A_2_ & dopamine D_2_ receptors in the striatum (for full discussions see, for example, [3-8]). We and others have shown that adenosine A_1_ receptor activation in the rat striatum both *in vivo* [9-12] and *in vitro* [13-17] inhibits dopamine release in a concentration dependent manner. This inhibitory effect is reduced by pre-perfusion with the D_1_ receptor antagonist SCH 23390. Taken together, this data supports the existence of a direct inhibitory modulation of striatal dopamine release by A_1_ receptors and given the lack of anatomical evidence for D_1_-like receptors on dopaminergic terminals [18] an indirect inhibitory modulation of striatal dopamine release by D_1_-like receptors through feedback loops involving other neurotransmitters.

Dopamine-glutamate reciprocal modulations play a major integrative role in the striatum. Glutamate acts on two types of glutamatergic receptors, ionotropic glutamatergic receptors (NMDA, AMPA and kainate) and metabotropic glutamatergic receptors that are G-protein coupled. Ionotropic NMDA receptors are found postsynaptically on GABA neurons [19]. These receptors are also expressed presynaptically on dopaminergic terminals [20]. NMDA receptor activation has been shown to enhance stimulated dopamine release in slice preparations. This facilitating action was reversed by NMDA receptor antagonists and was resistant to TTX, indicating that the receptors being activated are presynaptically located [21, 22]. In contrast, previous voltammetric studies have shown that the activation of NMDA receptors inhibits dopamine release [23, 24]. Thus it seems that NMDA receptor activation can have both a facilitatory and inhibitory effect on dopaminergic transmission. Conversely dopamine has also been shown to modulate glutamate release. Dopamine D_2_-like receptors are involved in the presynaptic inhibition of glutamatergic transmission [25]. The general consensus is that the receptors involved in the control of glutamate release throughout the striatum belong to the D_2_-like [26], but not the D_1_-like receptor family. The presence and the function of D_1_-like receptors on corticostriatal terminals is still a matter of some debate. A_1_ receptors located on corticostriatal terminals inhibit transmitter release through the blockade of Ca^2+^ currents [27]. As A_1_ receptors are located on glutamatergic terminals, it has been suggested that the ability of A_1_ receptors to modulate dopamine release is secondary to their ability to decrease glutamate release, resulting in a decrease in the activation of ionotropic glutamate receptors localized in dopaminergic terminals [12]. In the first section of the present study we investigate the role that NMDA receptor activation plays in the modulation of dopamine release and the influence of adenosine A_1_ receptors in this modulation.

GABA plays a central role in the processing of information in the striatum. There are two neuronal sources of GABA in the striatum, spiny projection neurons and intrinsic GABAergic interneurons. The spiny projection neurons are the prinicipal efferent cells of the striatum. These neurons receive excitatory input from motor cortices and thalamus and dopamine input from midbrain dopamine cells. Dopaminergic input is critical for the control of movement by the basal ganglia; its loss leads to the motor deficits observed in Parkinson's disease. Dopamine D_1_-like receptors are present on striato-nigral medium spiny output GABAergic neurons. Conversely striato-pallidal GABAergic output neurons express D_2_-like receptors [28, 29]. Localization of D_2_ and D_5_ receptors has also been demonstrated on GABAergic interneurons [1]. Dopamine enhances GABA release [30] and D_1_-like receptors have been implicated in this effect [31-33]. Evidence about the effect of GABA on dopamine transmission is conflicting. In vitro studies using fast cyclic voltammetry suggest that GABA enhances dopamine release, as blockade of striatal GABA_A_ receptors with picrotoxin caused a decrease in evoked dopamine release [34]. Other studies have also shown that GABA potentiates potassium stimulated ^3^H-dopamine release from striatal slices but did not alter spontaneous release [35]. However other in vitro studies suggest that GABA inhibits dopamine release [36-38]. In vivo studies have shown that perfusion of picrotoxin directly into the caudate nucleus, resulted in an increase in dopamine release [39]. Adenosine A_1_ receptors have been reported to be present on GABAergic terminals, where A_1_ receptor activation has been shown to be inhibitory [40]. With this in mind, the effect of GABA and adenosine receptor activity on dopamine release was also investigated.

## 2. Experimental Section

### 2.1 Brain slices

Male Wistar rats (50-75g) were killed by decapitation. The brain was quickly removed into ice-cold artifical cerebrospinal fluid (aCSF). Blocks of tissue containing the caudate putamen and nucleus accumbens were prepared. 350 μm thick slices were sectioned using a Campden vibrotome. Brain slices were then transferred to a holding chamber containing aCSF (see below) at room temperature (20-21°C) to equilibrate for 1 h. A single slice was then transferred to a recording chamber and perfused with oxygenated aCSF at 5 ml.min^-1^ at 30°C for 1 h before electrical stimulation.

### 2.2 Measurement of endogenous dopamine release

**Figure 1.**
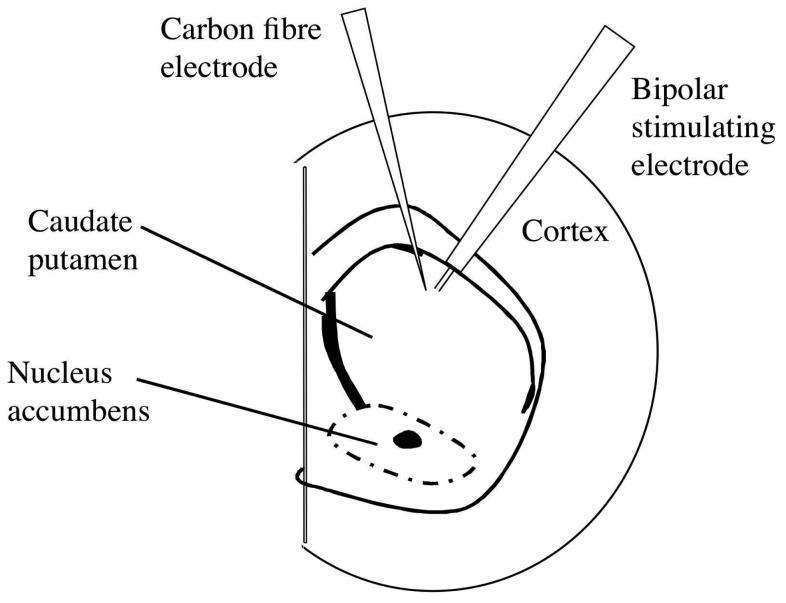
Placement of electrodes Schematic diagram illustrating the placement of the carbon fibre microelectrode (recording electrode) and the bipolar stimulating electrodes (tip separation 200 μm) in the dorsolateral striatum. The carbon fibre electrode was placed 100 to 200 μm from the bipolar stimulating electrodes.

**Figure 2.**
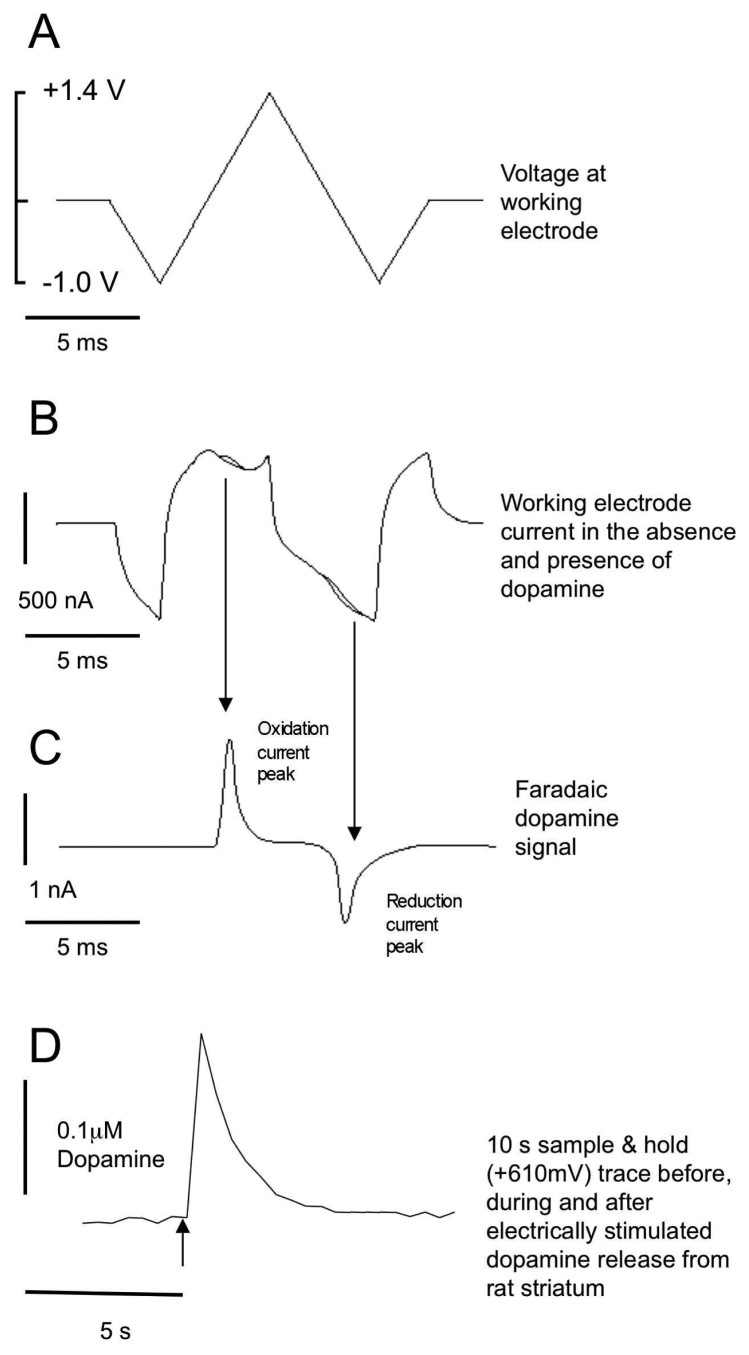
Waveforms used in FCV A. A triphasic voltage ramp is passed down the carbon fibre electrode twice per second (2 Hz). The ramp sweeps from 0 V (relative to silver/silver chloride reference electrode) to -1.0 V to +1.4 V to -1.0 V and back to 0 V. This sweep lasts 20 ms. B. The resultant current measured by the carbon fibre electrode in called the charging current. Superimposed on the charging current is the current obtained when the electrode is placed in a ringer solution containing 1 μM dopamine. C. If the charging current in B in the absence of dopamine is subtracted from that current in the presence of dopamine a trace typical of C is the result (subtractogram). This is known as the faradaic current and is the result of the oxidation and reduction of dopamine on the surface of the carbon fibre electrode. Dopamine oxidizes at approximately +610 mV and is reduced at approximately -200 mV. D. The trace illustrated in D is the result of a sample and hold device measuring at +610 mV during the electrical stimulation of the striatum. The arrow indicates the time of stimulation of striatum (0.1 ms pulse width; 10 V). Post calibration of the CFMe indicated that approximately 0.1μM dopamine is evoked by a single electrical stimulation in the dorsolateral striatum. Peak rise time is approximately 1 s and half decay time approximately 1.5 s.

Following 1 h equilibration, a bipolar tungsten-stimulating electrode with a tip separation of 200 μm (A-M Systems, Inc.) was placed in the dorsolateral CPu (see figure 1). A carbon fibre electrode (CFE; 7 μm diameter carbon fibre; 50-100 μm exposed length) was placed 100-200 μm from the stimulating electrode. In these set of experiments we manufactured the CFEs by hand and mechanically broke back the fibre to 50 - 100 μm from the glass seal (by a fine forceps) rather than spark etch the eletrodes (see reference [41] for more details on carbon fibre manufacture). Fast cyclic voltammetry (FCV; Millar Voltammeter; Dr. Julian Millar, Queen Mary & Westfield College, University of London, UK) at the CFE was used to detect changes in extracellular concentrations of dopamine following electrical stimulation of the brain slice [17, 42]. A triphasic voltage waveform (ranging from -1 to +1.4V; 20 ms duration), generated using a *Millar* voltammeter [43] was applied to the CFE at 2 Hz (every 500 ms; figure 2A). A sample and hold device built into the Millar voltammetry monitored dopamine release at +610 mV during each scan (figure 2B, C; [44]. Stimulated dopamine release (using *Neurolog* modules) was evoked using a square-wave pulse of 10 V amplitude and 0.1 ms duration delivered once every 5 min. A sample and hold output before during and after a stimulus is shown in figure 2D. Dopamine release under these conditions is tetrodotoxin-sensitive and Ca^2+^-dependent [42, 45]. Working voltage, faradiac current and sample and hold data was recorded onto a PC via a 4 channel *MacLab*.

### 2.3 Electrode calibration

**Figure 3.**
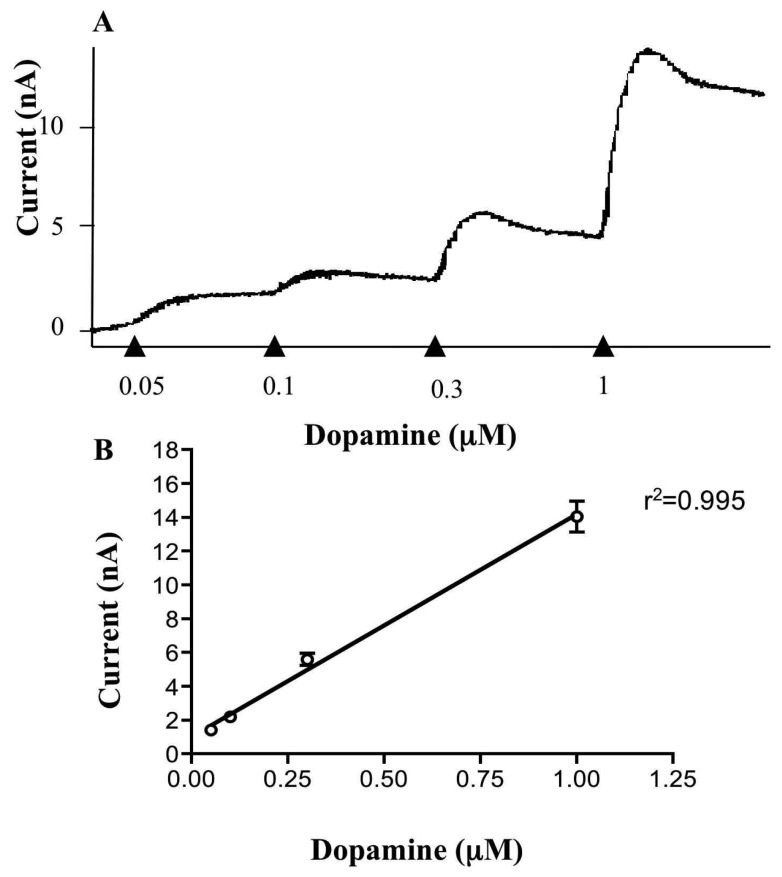
Calibration of the carbon fibre electrode Carbon fibre electrodes were routinely placed in known concentrations of freshly prepared dopamine after experiments in brain slices. A. Typical sample and hold output from an electrode after cumulative concentrations of dopamine were added (0.05 to 1 μM). The concentrations used are within the range of dopamine levels measured upon electrical stimulation in the striatum. B. Calibration curve showing the average of 20 carbon fibre electrodes. The relationship between dopamine concentration at the surface of the electrode and the measured current was found to be linear at these concentrations (r^2^ =0.9952).

A typical sample and hold trace (+610mV) obtained during the calibration of a carbon fibre electrode is shown in figure 3A. Electrodes were calibrated with increasing concentrations of freshly prepared dopamine in the range 0.05 μM to 1 μM, concentrations in the range of the endogenous dopamine released in the slices. An average standard calibration curve (n=20) in shown in figure 3B where the relationship of the dopamine concentration (μM) and the measured faradaic current (nA) was found to be linear in this range (r^2^=0.9952, linear correlation analysis).

### 2.4 Buffers and Drugs

**Figure 4.**
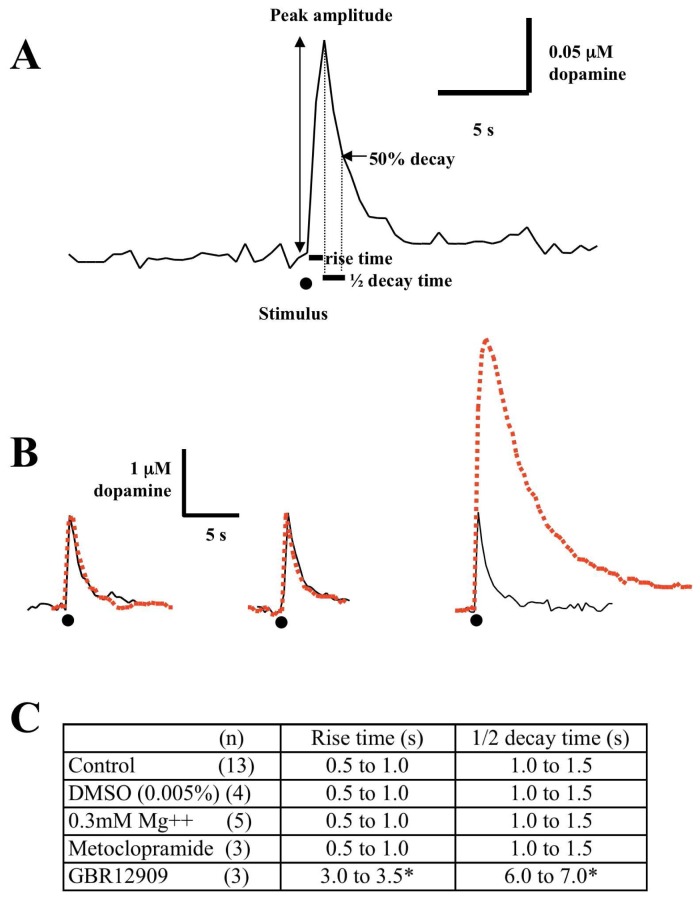
Effect of low Mg^2^^+^, metoclopramide and GBR 12909 on single pulse evoked dopamine release Single pulse evoked dopamine release was measured as the peak release in response to electrical stimulation. A. Rise time was measured from the beginning of the baseline to peak amplitude and half decay time was measured from peak release to 50% half decay. Dot indicates point of stimulation. B. Sample traces showing control stimulated dopamine release (black) and superimposed traces (all red) obtained after 30 min perfusion with 0.3 mM Mg^2+^ (left), 0.3 μm metoclopramde (centre) and 3 μM GBR12909 (right). Whilst low Mg^2+^ and metoclopramide have no effect on single pulse release, GBR12909 has a dramatic affect. C. Summary of the effects shown in B (*P<0.05).

Artificial cerebrospinal fluid (aCSF) was prepared every day according to the following composition in mM: NaCl, 120; KCl, 2.5; MgSO_4_, 2; CaCl_2_, 2; NaH_2_PO_4_, 1.25 and D-glucose, 10 mM in H_2_O. aCSF was bubled with 95% O_2_/5% CO_2_. N^6^-cyclopentyladenosine (CPA), isoguvacine hydrochloride, and metoclopramide monohydrochloride, were obtained from Sigma, UK and dissolved in de-ionized water. 8-cyclopentyl-1,3-dipropylxanthine (DPCPX; Tocris Cookson, UK) and picrotoxin (Sigma, UK) were dissolved in dimethylsulphoxide (DMSO). Stock solutions of DMSO were made to obtain concentrations of DMSO lower than 0.005% in the superfusing aCSF. Control experiments for solvent were carried out in parallel. This final concentration of DMSO (0.005%) in the perfusing aCSF did not affect single pulse dopamine release, rise time or decay time (figure 4C). NMDA and GBR12909 (both Tocris Cookson, UK) were dissolved in de-ionized water. All drugs were made up to stock solutions, which were stored at -80°C. All drugs were maintained in the perfusion medium for the entire duration of each experiment. All control experiments were carried out intermittently between drug experiments. In pre-treatment experiments drugs were pre applied for either 30 or 60 min.

### 2.5 Data analysis

Single pulse evoked dopamine over flow was measured as the peak release in response to electrical stimulation (figure 4). Rise time and half decay time of dopamine release were measured in some of the experiments. Rise time was measured from the beginning of baseline to peak amplitude and half decay time was measured from peak release to 50% half decay (see figure 4A). Stimulated dopamine release was measured for 20 min (4 stimulations) prior to drug application and the average of these 4 values were taken as 100%. All values subsequent to these were represented as % control. All data was analysed using the GraphPad Prism, version 4.00 (GraphPad Software Inc. 2003) statistical analysis platforms. Data are presented as means±standard error of the mean (sem) of n independent experiments (different brain slices). Students t-test (paired and unpaired sampling where appropriate) was used where P<0.05 was considered significant. In figure 7 one-way ANOVA was used to compare data from the different concentrations of isoguvacine to those in the presence of DCPCX.

## 3. Results

### 3.1 Effect of low magnesium on single pulse evoked dopamine release

Under normal physiological conditions, NMDA receptors are blocked by Mg^2+^ ions. To unmask the NMDA effect on dopamine transmission, experiments were carried out using low Mg^2+^ aCSF (0.3 mM Mg^2+^). The rise time of single pulse evoked dopamine release was not significantly affected by perfusion with low Mg^2+^ aCSF (figure 4B (first trace) and C). Also the half decay time of the dopamine signal did not differ significantly from controls. As a comparison to these data we also tested the dopamine D_2_ receptor antagonist, metoclopramide, which is know to affect multiple pulse dopamine release but not single pulse (see reference [46]) and GBR12909, the dopamine reuptake inhibitor. Metoclopramide had no effect on rise time or half decay time. However as expected GBR12909 caused a 300% increase in rise time and a 450% increase in half decay time (figure 4B and C).

### 3.2 Role of A1 receptors in the NMDA modulation of dopamine release

**Figure 5.**
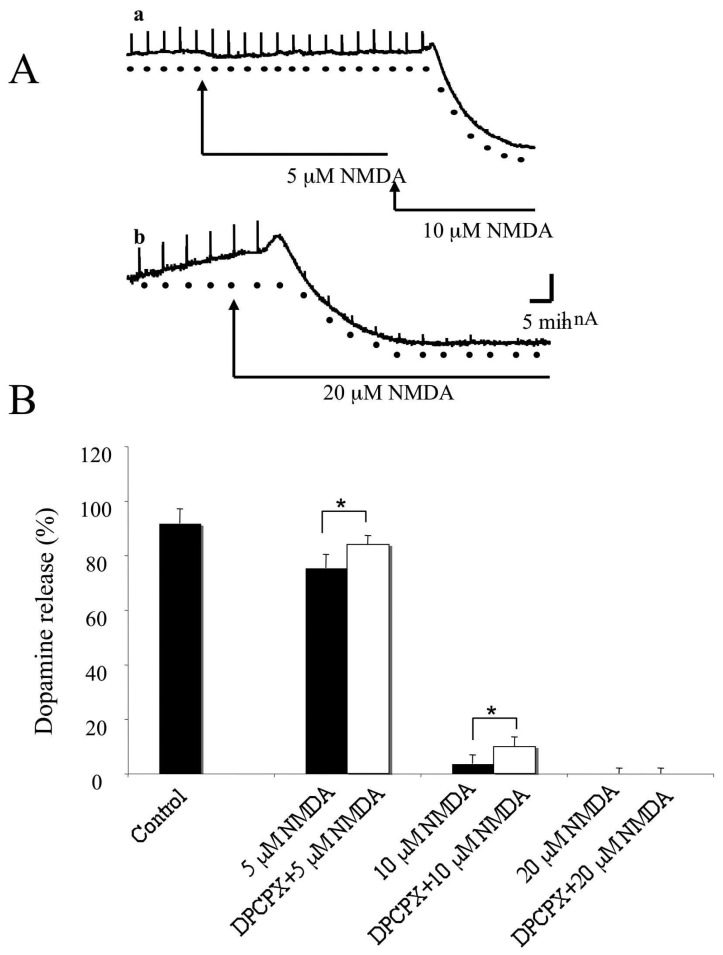
Effect of NMDA and DPCPX on single pulse evoked dopamine release A. Typical sample and hold data traces, showing the effects of three concentrations of NMDA on single pulse evoked dopamine release. Single pulse stimulation was applied at 5 min intervals. All experiments where carried out using low Mg^2+^ (0.3 mM in the aCSF). Perfusion with 5, 10 and 20 μM NMDA caused a concentration dependent inhibition of dopamine release. Perfusion with 20 μM NMDA caused a near complete inhibition of dopamine release in 5 out of 5 experiments. B. Summary graphs showing the effect of DCPCX (1 μM) on the inhibition of dopamine release by 5, 10 and 20 μM NMDA. Prior perfusion with DPCPX for 30 min before the application NMDA significantly attenuated the effects of 5 and 10 μM NMDA but not that of 20 μM NMDA (*P<0.05; see text for details).

Perfusion with NMDA (5, 10 and 20 μM) in low Mg^2^ caused a concentration dependent reduction of single pulse evoked dopamine release (control, 91.7±5.3%; 5 μM NMDA, 75.4±4.9%; 10 μM NMDA 3.6±3.1%; 20 μM NMDA, 0%; n=6-7; all at 60 min; figure 5A and B). Dopamine release was unaffected by perfusion with 1 μM DPCPX for 60 min (n=6). Pre-perfusion with DPCPX partially but significantly attenuated the inhibitory effect of both 5 and 10 μM NMDA on dopamine release (83.9±3.4 versus 75.4±4.9% and 9.8±3.6% versus 3.6±3.1%;, n=6, P<0.05 for both; figure 5B).

### 3.3 Role of the GABA_A_ receptor antagonist picrotoxin, in the CPA-induced inhibition of dopamine release

**Figure 6.**
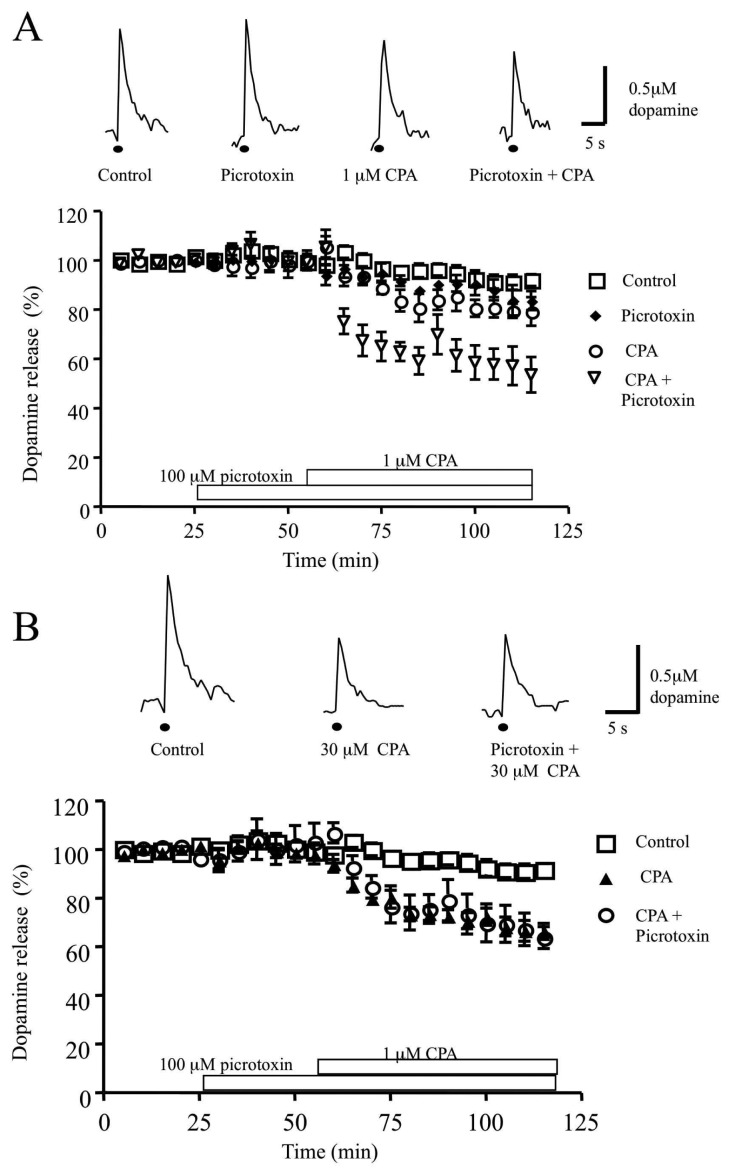
Picrotoxin enhances the inhibitory effect of 1 μM CPA but not 30 μM CPA on dopamine release A. Perfusion with 100 μM picrotoxin for 60 min did not significantly alter electrically evoked dopamine release (83.5±3.9%, n=5, P>0.05). However perfusion with 1 μM CPA reduced dopamine release compared to control values (79.3±5.8%, n=6, P>0.05). Prior perfusion with 100 μM picrotoxin significantly enhanced the inhibitory effect of 1 μM CPA on dopamine release (53.1±7.2%, n=6, P<0.01). Typical sample and hold traces are shown for controls, slices treated with 100 μM picrotoxin, 1 μM CPA and picrotoxin+CPA. All traces were obtained 60 min after drug application. Dots indicates point of stimulation of brain slice. B. Picrotoxin does not increase further the inhibitory effect of 30 μM CPA on dopamine release. Addition of 30 μM CPA for 60 min significantly inhibited dopamine release (control, 92±2.3%, n=13 versus 30 μM CPA, 66.5±3.3%, n=5). Prior perfusion with 100 μM picrotoxin for 30 min did not alter the effect of CPA when compared to CPA alone (63.8±4.5, n=5). Typical sample and hold traces were obtained 60 min after drug application for control, 30 μM CPA and picrotoxin+CPA.

Perfusion with picrotoxin (100 μM) for 60 min did not alter single pulse evoked dopamine release (control, 91.6±2.3%, n=13 versus picrotoxin, 83.5±3.9%, n=5, P>0.05). This may imply that GABA_A_ receptors do not tonically modulate dopamine transmission, at least in the in vitro preparation. Addition of the adenosine A_1_ receptor agonist, CPA at 1 μM (this concentration has previously been shown to cause moderate inhibitory effects on dopamine release [17]) caused a small but significant reduction in dopamine release compared to control values (79.3±5.8%, n=6, P<0.05). However the inhibition of dopamine release by CPA was significantly enhanced by prior perfusion with picrotoxin for 30 min before the addition of CPA (53.1 ±7.2%, n=6, P<0.01; figure 6A). Perfusing the slice with a higher concentration of CPA (30 μM; previously shown to cause near maximal effects on dopamine release, [17]) for 60 min also caused a significant reduction in dopamine release (control, 92±2.3%, n=13 versus 30 μM CPA 66.5±3.3%, n=5, P<0.001; figure 6B). However prior perfusion with picrotoxin (100 μM) for 30 minutes did not significantly increase this inhibitory effect of 30 μM CPA on dopamine release (63.8±4.5%, n=5, P>0.05). Indeed a maximum inhibitory effect may already have been reached at this concentration of CPA (see reference [17]).

### 3.4 Role of the GABA_A_ receptor agonist, isoguvacine and the A_1_ receptor antagonist DPCPX in dopamine release

**Figure 7.**
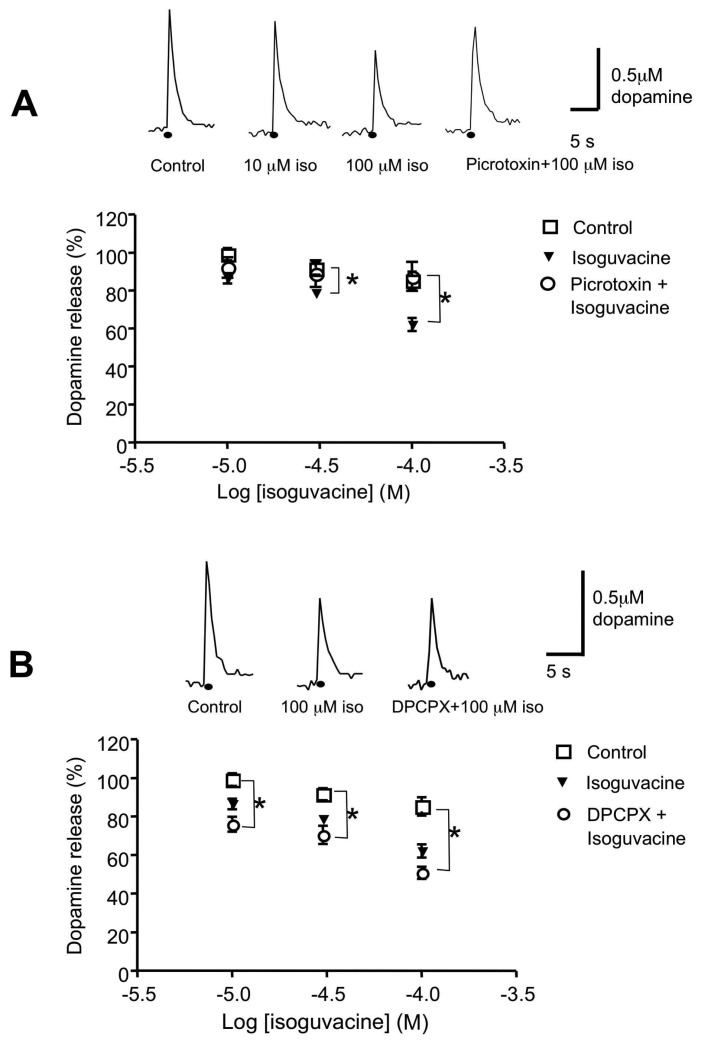
The inhibition of dopamine release in the rat striatum by isoguvacine is modulated by DPCPX A Concentration response curve for the effects of isoguvacine on dopamine release. Results are expressed as dopamine release (%), 30 minutes after perfusion with each cumulative concentrations of isoguvacine (10, 30 and 100 μM), versus log concentration of isoguvacine. Isoguvacine inhibited dopamine release in a concentration-dependent manner, that reached significance 30 min after perfusion (e.g. control 85.2±4.9%, n=7 versus 100 μM isoguvacine, 62.1±3.4%, n=5. P<0.05). Pre treatment of slices with picrotoxin reversed the inhibitory effects of 100 μM isoguvacine (100 μM isoguvacine, 50.8±3.1%, n=5 versus picrotoxin+100 μM isoguvacine, 87.4±7.6%, n=5, P<0.05). Shown are typical sample and hold traces obtained under control conditions and 30 min after the perfusion of 10 μM isoguvacine, 100 μM isoguvacine and picrotoxin+isoguvacin. B. The inhibition of dopamine release by isoguvacine is modulated by prior application of the adenosine receptor antagonist DPCPX. In the presence of DPCPX, isoguvacine significantly inhibited dopamine release at all concentrations compared to control values. (DPCPX+10 μM isoguvacine, 75.9±3.8%, n=5, P<0.01 versus controls; DPCPX+30 μM isoguvacine, 70.4±4.6%, n=5, P<0.05 versus controls; DPCPX+100 μM isoguvacine, 50.8±3.1%, n=5. P<0.001 versus controls). Using one-way analysis of variance these effects were found to be significantly different from treatment with isoguvacine alone (P<0.05). Shown are typical sample and hold traces obtained under control conditions, and 30 min after the perfusion of 100 μM isoguvacine and DPCPX+ isoguvacine. Dots indicate point of stimulation in the slice.

Perfusion of cumulative concentrations of isoguvacine (1, 10, 30 and 100 μM), the selective GABA_A_ receptor agonist, inhibited dopamine release in a concentration dependent manner. Results are expressed as dopamine release versus the log concentration of isoguvacine in figure 7A. Perfusion with 1 or 10 μM isoguvacine did not significantly reduce dopamine release (e.g. control, 99.1±3.3%, n=7; 10 μM isoguvacine, 86.4±2.8, n=5; P>0.05). However perfusion with 30 or 100 μM isoguvacine, for 30 min significantly inhibited dopamine release (78.8±2.1 and 62.1±3.4%, respectively; n=5, P<0.05; figure 7A). Prior perfusion with picrotoxin (100 μM) for 30 min significantly reduced the inhibitory effect of 30 and 100 μM isoguvacine on dopamine release (e.g. 100 μM isoguvacine, 62.1±3.4% vs picrotoxin+100 μM isoguvacine, 87.4±7.6% n=5, P<0.05).

The role played by A_1_ receptors in the inhibitory effect of isoguvacine on dopamine release was investigated. Slices were pre-perfused with the A_1_ receptor antagonist, DPCPX (1 μM) for 30 min before addition of isoguvacine. In the presence of DPCPX, the inhibitory effect of isoguvacine on dopamine release was significantly enhanced (e.g. DPCPX+10 μM isoguvacine, 75.9±3.8%, n=5, P<0.01 versus controls; DPCPX+30 μM isoguvacine, 70.4±4.6%, n=5, P<0.05 versus controls; DPCPX+100 μM isoguvacine, 50.8±3.1%, n=5. P<0.001 versus controls). Using one way analysis of variance the inhibitory effect of isoguvacine in the presence of DPCPX on dopamine release was found to be significantly greater than treatment with isoguvacine alone (F=5.1; P<0.05; Figure 7B).

## 4. Discussion

In the present studies we have shown that the inhibitory effects of NMDA on electrically stimulated dopamine release can be modulated by adenosine (A_1_) receptor block and that the inhibitory effect of adenosine (A_1_) receptor activation on electrically stimulated dopamine release can be enhanced by GABA receptor block. In addition we also demonstrate a role for A_1_ receptors in the inhibitory effects of isoguvacine (GABA agonist) on electrically stimulated dopamine release.

### 4.1 Glutamate transmission

Electrical stimulation of the dorsal striatum releases many neurotransmitters including dopamine, glutamate, acetylcholine, adenosine and GABA. NMDA receptor activation has been shown to increase adenosine release in striatal slices [47], which in turn inhibits the release of excitatory amino acids via A_1_ receptors located on glutamatergic terminals. We therefore set out to look at any possible modulation of the inhibitory effects of NMDA on dopamine release by adenosine receptor activation. As demonstrated with previous voltammetric studies [23, 24] we have shown that perfusion of NMDA in low Mg^2+^ aCSF, inhibited electrically evoked dopamine release. Low Mg^2+^ aCSF alone did not alter the release and uptake kinetics of dopamine release. Single pulse evoked dopamine release was very sensitive to NMDA receptor activation. Perfusion with concentrations of NMDA of 10 and 20 μM near completely abolished stimulated dopamine release. Irvani & Kruk (1996), proposed that, activation of NMDA receptors causes a large release of dopamine, leading to a depletion of dopamine, and subsequent inhibition of electrically stimulated dopamine release. In our experiments using 10 and 20 μM NMDA, a transient increase in the charging current, followed by a decrease in the charging current was observed, as has been reported by [23], suggesting that perfusion with NMDA does alter the components that contribute to the charging current and possibe a depletion of dopamine. Application of NMDA is likely to lead to global depolarisation of the tissue an effect independent of the presence of NMDARs on dopamine axons. This would deplete the releasable pool of dopamine and give rise to the inhibition of stimulated dopamine release that we have observed.

Pre-perfusion with 1 μM DPCPX, the adenosine A_1_ receptor antagonist, for 30 min had a small but significant attenuating effect on the effects of 5 μM NMDA on dopamine release. 1 μM DPCPX has been previously used in studies in the nucleus accumbens [48]. Lower concentrations of 100 to 200 nM have also been used (see for example the effects of DPCPX on synaptic plasticity in the hippocampus [49]. Pre-perfusion with DPCPX did also reduce albeit to a small degree, the large inhibitory effect, of 10 μM NMDA on dopamine release. These results are hard to interpret as NMDA may be depleting neurones of dopamine. Adenosine also plays a major role in hypoxia and tissue damage in slices [50] and the attenuating efects of adenosine receptor block on the NMDA inhibition of dopamine release may involve many of these signalling mechanisms. Although adenosine does have a significant modulating role in both glutamatergic and dopaminergic transmission, in our experiments it does not seem to play a major role in the glutamatergic modulation of dopamine release.

### 4.2 GABA transmission

We next investigated a role for GABARs in the adenosine-induced inhibition of dopamine release. Perfusion with 100 μM picrotoxin, at a concentration that has been used previously in brain striatal slices [45, 34], did not alter single pulse evoked dopamine release. This is in agreement with previous in vitro voltammetric experiments using GABAzine to block GABA_A_ receptors, indicating a lack of GABAergic tone in the slice preparation [51]. In our experiments picrotoxin significantly enhanced the inhibitory effect of the lower concentration of CPA (1 μM) on dopamine release but had no effect on the higher concentration of CPA (30 μM). These results are surprising because, if picrotoxin was acting on GABA_A_ receptors located on dopamine terminals one would expect an increase in dopamine release. In vivo microdialysis studies have shown that bicuculline, a GABA_A_ receptor antagonist, increased striatal acetylcholine output, whereas muscimol, a GABA_A_ receptor agonist, decreased striatal acetylcholine output [52]. It may be possible that picrotoxin is acting on GABA_A_ receptors located on acetylcholine interneurons [53], enhancing acetylcholine release. Prior perfusion with picrotoxin did not alter the inhibitory effect of the higher concentration of CPA (30 μM). Perfusion with 30 μM CPA reduced dopamine release to 66.5±3 %, which is not significantly different to the inhibitory effect of perfusion with 100 μM CPA (56.8±10%; results not shown). This may imply that perfusion with 30 μM CPA elicits maximum inhibition in this system and addition of picrotoxin did not increase the maximal effect of CPA.

Perfusion with isoguvacine, the GABA_A_ receptor agonist inhibited dopamine release in a concentration-dependent manner, which was reversed by pre-perfusing the slice with picrotoxin, suggesting that isoguvacine is acting directly via GABA_A_ receptors to inhibit evoked dopamine release. A high concentration of isoguvacine was required to significantly inhibit dopamine release. However in vitro patch clamp studies carried out in brain slices containing the ventral tegemental area used concentrations that ranged from 10-300 μM isoguvacine and they obtained an EC_50_ value of 62±8 mM isoguvacine [54]. Using the post-embedding immunogold technique on freeze-substituted tissue, Fujiyama et al., 2000 [55] were unable to rule out the presence of GABA_A_ receptors on dopamine terminals. Also GABA activity has been shown to modulate dopamine transmission, possibly by acting through GABA_A_ receptors on nigrostriatal dopaminergic terminals [36, 37, 56]. Dopamine D_1_-like receptors found on the GABAergic neurons of the direct pathway and also fast-spiking GABAergic interneurons, depolarize the cell membrane and enhances electrically evoked GABA release [1, 57]. The attenuation of inhibitory GABAergic tone on dopaminergic neurons results in increased dopamine release. The A_1_ receptor antagonist, DPCPX, enhanced the isoguvacine effect on dopamine, an effect which was significant when compared to isoguvacine alone. DPCPX may be acting on A_1_ receptors found on GABAergic terminals and may produce an overall increase of GABA in the synaptic cleft, thus enhancing the isoguvacine effect. D_2_-like receptors are located on GABAergic interneurons and striatopalidal spiny projection neurons, were they inhibit GABA release [33, 58]. A role for D_2_ receptor activation in the effects of CPA on dopamine release was ruled out, as metoclopramide has previously been shown not alter the CPA effect; see reference [17].

In the in vitro slice preparation, connectivity between different neurons is likely to be at least partially maintained. It is possible that ligands are acting on receptors other than those found on nigrostriatal terminals to modulate neurotransmitter release. These neurotransmitter systems interact with each other and with voltage-dependent conductances to regulate dopaminergic transmission. The dopamine response in our experiements is elicited by a short, discrete stimulus, so although other neurotransmitters are being released, it is unlikely that changes in neurotransmitter levels due to electrical stimultion can act on the evoked dopamine. The dopaminergic terminals contain fast acting ionotropic receptors and it is likely that the concentration of neurotransmitters in the synaptic cleft and extracellular space plays a fundamental role in determining the excitability of the dopaminergic terminal, thus determining the amount of dopamine released due to a single pulse stimulus.

## Conclusion

In conclusion in our experiments using the technique of fast cyclic voltammetry, we have demonstrated a novel role for adenosine, glutamate and GABA in the modulation of dopamine release in the dorsolateral striatum.
